# Glecirasib treatment in *KRAS G12C*-positive non-small cell lung cancer with leptomeningeal metastasis

**DOI:** 10.3389/fonc.2026.1956344

**Published:** 2026-09-23

**Authors:** Yunfei Li, Junping Wei, Mohan Li, Ting Xu, Shencun Fang, Na Liu

**Affiliations:** 1 Department of Respiratory Medicine, The People’s Hospital of Linquan County, Fuyang, China; 2 School of Clinical Medicine, Jiamusi University, Jiamusi, Heilongjiang, China; 3 Brain Metastases Diagnosis and Treatment Centre, The Affiliated Brain Hospital of Nanjing Medical University, Nanjing, China; 4 Department of Respiratory Medicine, Nanjing Chest Hospital, The Affiliated Brain Hospital of Nanjing Medical University, Nanjing, China

**Keywords:** cerebrospinal fluid, circulating tumor DNA, glecirasib, KRAS G12C, leptomeningeal metastasis, non-small-cell lung cancer

## Abstract

**Background:**

Leptomeningeal metastasis (LM) is associated with poor outcomes in non-small cell lung cancer (NSCLC), and clinical evidence for *KRAS G12C* inhibition in this setting is limited. We examined clinical outcomes and cerebrospinal fluid (CSF) molecular changes in patients with *KRAS G12C*-positive LM who received glecirasib.

**Methods:**

This retrospective case series included patients treated with glecirasib at Affiliated Brain Hospital of Nanjing Medical University between January 2025 and June 2026. Intracranial progression-free survival (iPFS) and overall survival (OS) were summarized descriptively. Exploratory serial CSF circulating tumor DNA (ctDNA) analyses were performed in two selected patients.

**Results:**

Seven patients were included, with a median observed follow-up of 6.3 months (range, 2.4-9.6). The best overall LM assessment was response in five patients, stable disease in one, and progressive disease in one. Two patients developed LM progression and one patient died; median iPFS and OS were not reached. In the patient with acquired *KRAS G12C*, CSF ctDNA detected the alteration at LM progression after EGFR-TKI therapy. *KRAS G12C* variant allele frequency decreased after glecirasib initiation and subsequently increased at progression, accompanied by the emergence of *KRAS G12D* and increased *CDK4/MDM2* amplification. In the patient with primary *KRAS G12C*, *KRAS G12C* and *TP53* variant allele frequencies initially decreased but later rose at the last CSF assessment, without concurrent radiographic, cytological, or symptomatic evidence of LM progression.

**Conclusions:**

In this retrospective series of seven patients, glecirasib monotherapy was associated with LM disease control in six patients, suggesting possible activity in KRAS G12C-positive NSCLC with LM.

## 1 Introduction

Leptomeningeal metastasis (LM) results from tumor dissemination within the leptomeninges and cerebrospinal fluid (CSF) and can cause neurological dysfunction at multiple levels of the neuraxis in patients with non-small cell lung cancer (NSCLC) (1). Evaluating treatment response is difficult because neurological findings, CSF cytology, and magnetic resonance imaging may not change in parallel, a limitation reflected in the proposed RANO-LM response criteria (2). Although targeted therapies have changed the management of LM in some molecularly selected NSCLC populations (3, 4), much of the available molecular evidence has focused on EGFR-mutant disease (5). Clinical data for patients with KRAS G12C-positive NSCLC and LM remain sparse.

Glecirasib is an orally administered covalent KRAS G12C inhibitor with documented systemic activity and manageable safety in advanced KRAS G12C-mutant NSCLC (6). In a mouse model of intracranially implanted KRAS G12C-mutant lung cancer, glecirasib reduced brain tumor bioluminescent signals (7). This result provides a rationale for investigating its activity in patients with central nervous system(CNS) involvement, but the model did not reproduce LM or establish drug exposure within the CSF. The clinical activity of glecirasib in KRAS G12C-positive LM therefore remains uncertain.

In this retrospective case series, we describe seven patients with KRAS G12C-positive NSCLC and LM who received glecirasib. LM response was evaluated using neurological, symptomatic, cytological, and imaging findings. Intracranial progression-free survival, overall survival, and safety were summarized descriptively. Serial CSF circulating tumor DNA was available for two patients: one with acquired KRAS G12C after EGFR-TKI therapy and one with primary KRAS G12C-mutant NSCLC. These longitudinal samples were examined to describe molecular changes during treatment rather than to establish the predictive performance of CSF ctDNA.

## 2 Materials and methods

### 2.1 Study design and patients

This retrospective case series was conducted at the Affiliated Brain Hospital of Nanjing Medical University and involved consecutive patients with *KRAS G12C*-positive NSCLC who developed LM and received glecirasib between January 2025 and June 2026. Patients were included in this study if they met all of the following criteria: (1) histologically or cytologically confirmed NSCLC; (2) molecular evidence of *KRAS G12C* detected in tumor tissue, plasma, CSF, or another clinically validated specimen; (3) LM diagnosed on compatible neurological manifestations together with positive CSF cytology and/or contrast-enhanced CNS magnetic resonance imaging; (4) treatment with glecirasib 800 mg once daily after LM diagnosis, without concurrent CNS-directed anticancer therapy during glecirasib; and (5) available baseline and follow-up data for outcome assessment. Patients were excluded from the study if LM diagnosis could not be clinically supported, if glecirasib was not administered, or if no post-treatment follow-up information was available.

### 2.2 Assessment of leptomeningeal metastasis

Overall, LM response was assessed according to the RANO-LM criteria (2). Overall LM response was categorized using the following prespecified principles: improvement in at least one major LM domain without deterioration in the remaining domains and, for positive baseline CSF cytology, cytological clearance or clearly documented cytological improvement. For patients with negative baseline cytology, response could be assigned from concordant neurological/symptomatic and imaging improvement without a cytological conversion requirement. Stable disease was defined by mixed or discordant findings that do not meet criteria for response or progression. Finally, disease was considered progressive when indicated by neurological, cytological, or radiographic deterioration, or when LM-directed treatment was modified due to clinical progression. Assessments were obtained as part of routine care rather than at protocol-mandated intervals, and the integrated classification was based on treating-team documentation without blinded independent central review. Systemic disease response was assessed separately using extracranial imaging results recorded in routine clinical practice.

### 2.3 CSF ctDNA sequencing and molecular monitoring

Serial CSF ctDNA monitoring was not protocol mandated. The two longitudinal cases were included because repeated CSF samples had been obtained for clinical indications and yielded reportable cell-free DNA results; selection was therefore availability based and may introduce selection bias. CSF samples were collected during routine lumbar puncture or ventricular reservoir procedures. A certified third-party clinical laboratory was contracted to extract cell-free DNA from CSF supernatant for hybrid capture-based targeted next-generation sequencing. The samples were analyzed using a 1,150-gene solid-tumor pan-cancer panel. The assays detected single-nucleotide variants, insertions/deletions, copy-number alterations, rearrangements/fusions, tumor mutation burden, and microsatellite instability. Sequencing was performed using the MGISEQ-2000 platform with GRCh37/hg19 as the reference genome. The mean sequencing depth was assay-dependent: Targeted monitoring assays required at least 1,000×, whereas selected high-depth 1,150-gene monitoring samples used a threshold of at least 5,000×. According to the clinical laboratory specifications, the lower limit of detection was 0.5% variant allele frequency (VAF) for cell-free DNA-based liquid biopsy samples. The study relied on finalized clinical reports. Historical records did not consistently retain CSF volume, processing-to-storage interval, extraction kit, input mass, the exact assay tier used at each time point, read-level quality/strand-bias/deduplication filters, or the proprietary copy-number normalization algorithm and amplification threshold. Accordingly, all serial CSF ctDNA analyses were exploratory, and longitudinal VAF changes were interpreted descriptively rather than as precise measures of tumor burden or as validated predictors of progression.

### 2.4 Outcomes and statistical analysis

The main clinical outcomes were LM response, intracranial progression-free survival (iPFS), and overall survival (OS). iPFS was defined as the time from glecirasib initiation to documented LM or CNS progression, death from any cause, or last follow-up. OS was defined as the time from glecirasib initiation to death from any cause or last follow-up. The individual components of LM assessment were also described, including changes in neurological status and symptoms, CSF cytology, and CNS imaging findings.

Given the sample size and noncomparative design, all analyses were descriptive, and no formal hypothesis testing was performed. Individual iPFS and OS trajectories were displayed using swimmer plots. Exploratory Kaplan-Meier curves with numbers at risk were added for transparency, but confidence intervals and median estimates were not emphasized because only two iPFS events and one death occurred. Exploratory serial CSF ctDNA results were plotted over time to describe reported changes in variant allele frequency and copy-number alterations at the available sampling points.

## 3 Results

### 3.1 Patient characteristics and baseline LM features

As shown in **Table 1**, seven patients with KRAS G12C-positive LM received glecirasib. The median age was 63 years (range, 50–71 years), and five patients were male. All patients had lung adenocarcinoma. Six patients had primary KRAS G12C-mutant NSCLC, whereas one patient had EGFR exon 19 deletion-positive NSCLC with acquired KRAS G12C detected in CSF at LM progression. The median interval from initial NSCLC diagnosis to LM diagnosis was 20 months (range, 3–57 months), and the median ECOG performance status at glecirasib initiation was 3 (range, 2–4). Patients had received a median of two prior systemic treatment lines (range, 1–5), including prior KRAS G12C inhibitor therapy in three patients.

**Table 1:** Patient baseline characteristics.

Case	Age	Sex	Interval to LM (mo)	Driver at LM	Other alterations at LM	PD-L1 TPS	Prior KRAS G12C inhibitor	Prior CNS local therapy	Extracranial disease at LM
1	62	Female	33	EGFR 19del, KRAS G12C	None reported	1%	No	Yes	SD
2	71	Female	3	KRAS G12C	RAF1/TP53/TSC1	50%	Fulzerasib	Yes	SD
3	63	Male	57	KRAS G12C	TP53/CHD4/JAK3/CXCR4	55%	No	Yes	PD
4	71	Male	20	KRAS G12C	TP53/CDK4 amp	30%	No	No	SD
5	63	Male	3	KRAS G12C	SDHB/TP53	70%	Fulzerasib	No	SD
6	50	Male	12	KRAS G12C	TP53/EGFR amp	20%	Fulzerasib	Yes	SD
7	62	Male	22	KRAS G12C	None reported	45%	No	No	SD

CNS, central nervous system; LM, leptomeningeal metastasis; PD, progressive disease; SD, stable disease; TPS, tumor proportion score.

Detailed demographic characteristics, neurological manifestations, and prior treatment histories are provided in **Supplementary Table S1**. Baseline LM diagnostic findings are summarized in **Supplementary Table S2**. All seven patients underwent baseline CSF cytology: five had positive cytology together with MRI evidence of LM, whereas Cases 2 and 4 had MRI-defined LM with negative cytology; no patient had unevaluable cytology. MRI patterns were classified as linear in two patients, nodular in two, and mixed in three. Serial CSF ctDNA monitoring was performed in Cases 1 and 6. Serial CSF ctDNA monitoring was performed in Cases 1 and 6 on the basis of available clinically indicated serial samples rather than a prespecified molecular selection rule.

### 3.2 Clinical activity of glecirasib in LM

Glecirasib treatment was associated with an overall LM response in five patients, stable LM in one (Case 1), and progressive LM in one (Case 3). Case 1 had symptomatic and radiographic improvement but persistent positive CSF cytology and was therefore classified as stable disease. By contrast, Cases 2 and 4 had negative baseline cytology and were classified as responses on concordant clinical/neurological and imaging improvement without a cytological conversion requirement (**Supplementary Table S3**). Disease control at best response was observed in six of seven patients. No unequivocal extracranial progression was documented during glecirasib treatment; the best systemic response was stable disease in all seven patients.

Individual iPFS and OS are shown in **Figure 1**, and exploratory Kaplan-Meier curves are shown in **Supplementary Figure S1**. The median observed follow-up was 6.3 months (range, 2.4-9.6). Two patients developed LM progression. Case 3 progressed at 2.9 months and died of lung infection at 3.5 months; Case 1 developed spinal leptomeningeal progression at 6.3 months and was alive at last follow-up. The remaining five patients were progression-free at last follow-up, with ongoing iPFS values of 2.4-9.6 months. Thus, disease control at best response was 6/7, whereas 5/7 were progression-free at last follow-up. Among three patients previously exposed to fulzerasib (Cases 2, 5, and 6), all three had an LM response and remained progression-free. Among four KRAS G12C inhibitor-naive patients, two had a response, one had stable disease, and one had progressive disease. Treatment-related adverse events were documented in 6 of 7 patients. One patient developed grade 3 elevations in AST and ALT. No grade 4–5 treatment-related adverse events were recorded (**Supplementary Table S4**).

**Figure 1:**
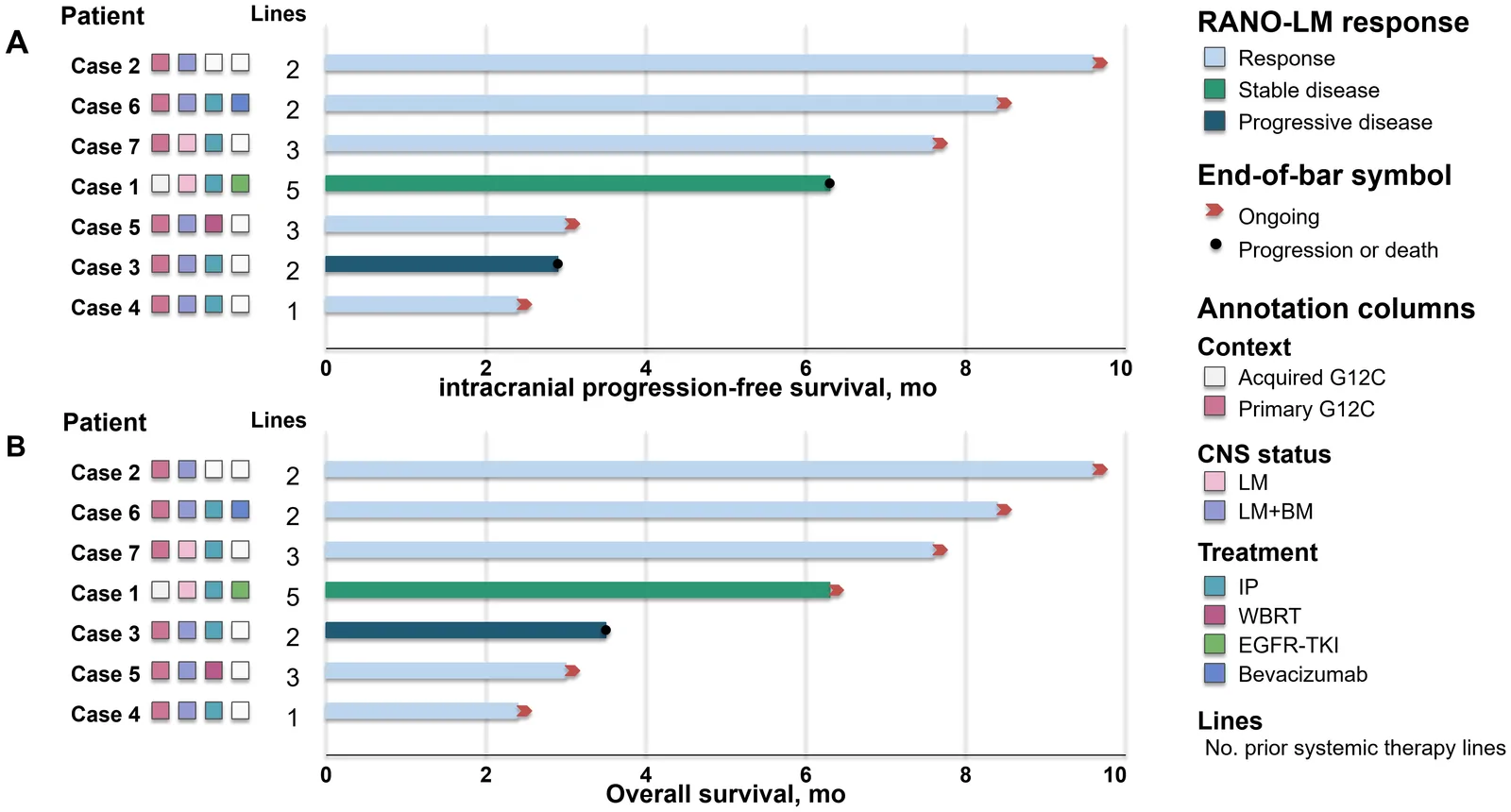
Individual intracranial progression-free survival **(A)** and overall survival **(B)** from glecirasib initiation. Bar color indicates best integrated RANO-LM assessment. A black circle denotes intracranial progression or death, and a red arrowhead denotes censoring/ongoing follow-up. Annotation columns show KRAS G12C context, baseline CNS status, and treatments received before glecirasib.

### 3.3 Serial CSF ctDNA monitoring in an acquired *KRAS G12C* case

Case 1 had *EGFR* exon 19 deletion-positive lung adenocarcinoma and had previously received surgery and multiple *EGFR*-TKI-based treatments. After symptomatic leptomeningeal progression in June 2025, the patient achieved symptom improvement with stable neurological assessment. LM progressed again, as indicated on MRI in December 2025. After CSF ctDNA revealed acquired *KRAS G12C*, glecirasib treatment was initiated. Contrast-enhanced MRI demonstrated regression of leptomeningeal enhancement, and symptoms improved; however, CSF cytology remained positive. This is why their best overall LM response was classified as stable disease. In June 2026, spinal leptomeningeal progression occurred, and glecirasib treatment was discontinued.

Serial CSF ctDNA provided a dynamic molecular view of treatment response and resistance evolution in this acquired *KRAS G12C* case (**Figure 2**). Before glecirasib initiation, CSF ctDNA detected *EGFR* exon 19 deletion with a VAF of 5.25%, consistent with persistent *EGFR*-driven CNS compartment disease. At LM progression in December 2025, *EGFR* exon 19 deletion increased to 18.64%, and *KRAS G12C* newly emerged at 10.81%, supporting the emergence of *KRAS G12C* as an acquired resistance alteration in CSF. After glecirasib initiation, *KRAS G12C* decreased to 1.59% by April 2026, in parallel with symptomatic and radiographic disease control. At the time of subsequent LM progression, CSF ctDNA showed higher reported VAFs, with *KRAS G12C* increasing to 3.42% and *KRAS G12D* increasing to 9.17% in June 2026, accompanied by increased *CDK4* and *MDM2* amplification.

**Figure 2:**
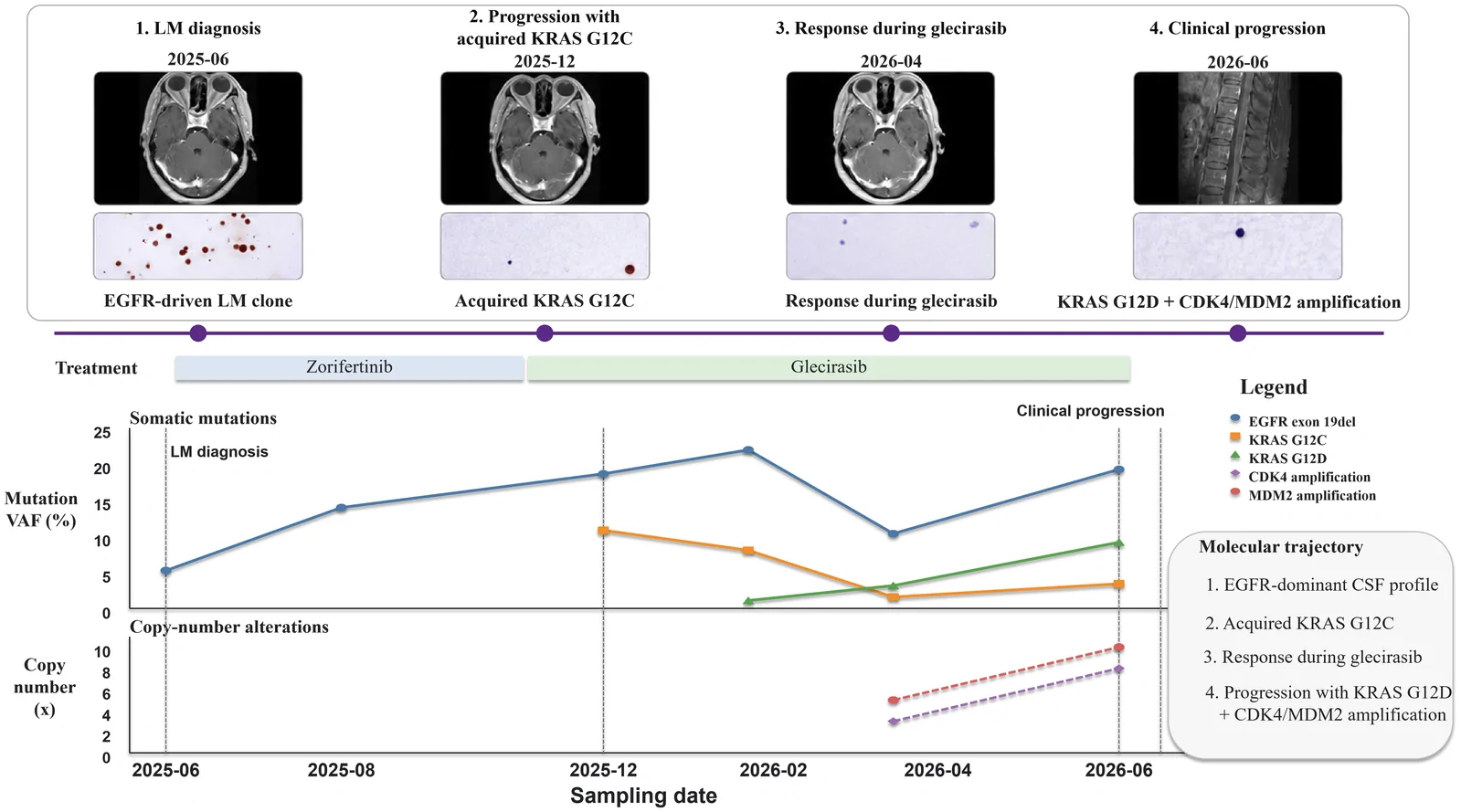
Acquired KRAS G12C case (Case 1): clinical, cytological, imaging, and CSF ctDNA trajectory. All molecular values were obtained from CSF-supernatant cell-free DNA reports. The exact historical assay tier at each time point was not recoverable; quantitative cross-time comparisons are descriptive.

### 3.4 Serial CSF ctDNA monitoring in a primary *KRAS G12C* case

Case 6 had primary *KRAS G12C*-mutant advanced lung adenocarcinoma. After initial chemoimmunotherapy, the patient received fulzerasib and achieved a PFS of 13 months. In September 2025, brain metastases and LM were diagnosed after the patient experienced headache, dizziness, and gait instability. Glecirasib was initiated after LM diagnosis. The overall situation was assessed as a response, and glecirasib was continued at the last follow-up.

Serial CSF ctDNA showed an initial decrease in the reported VAFs followed by an increase at the final CSF assessment (**Figure 3**). At baseline, CSF ctDNA showed a reported *KRAS G12C* VAF of 70.07%, accompanied by a *TP53 G244fs* VAF of 58.74% and *EGFR* amplification with a reported copy number of 10. After glecirasib treatment, the reported *KRAS G12C* VAF decreased to 19.63% and then to 8.97%, while the reported *TP53 G244fs* VAF decreased to 32.35% and then to 1.72%. *EGFR* amplification, detected only at baseline, was not detected in subsequent CSF assessments. In June 2026, the reported *KRAS G12C* and *TP53 G244fs* VAFs increased to 17.03% and 12.57%, respectively. The clinical significance of these VAF increases is uncertain. Glecirasib was continued because no concurrent clinical, MRI, or cytological evidence of LM progression was present at that time. This molecular-clinical discordance is an exploratory observation that requires validation in larger studies.

**Figure 3:**
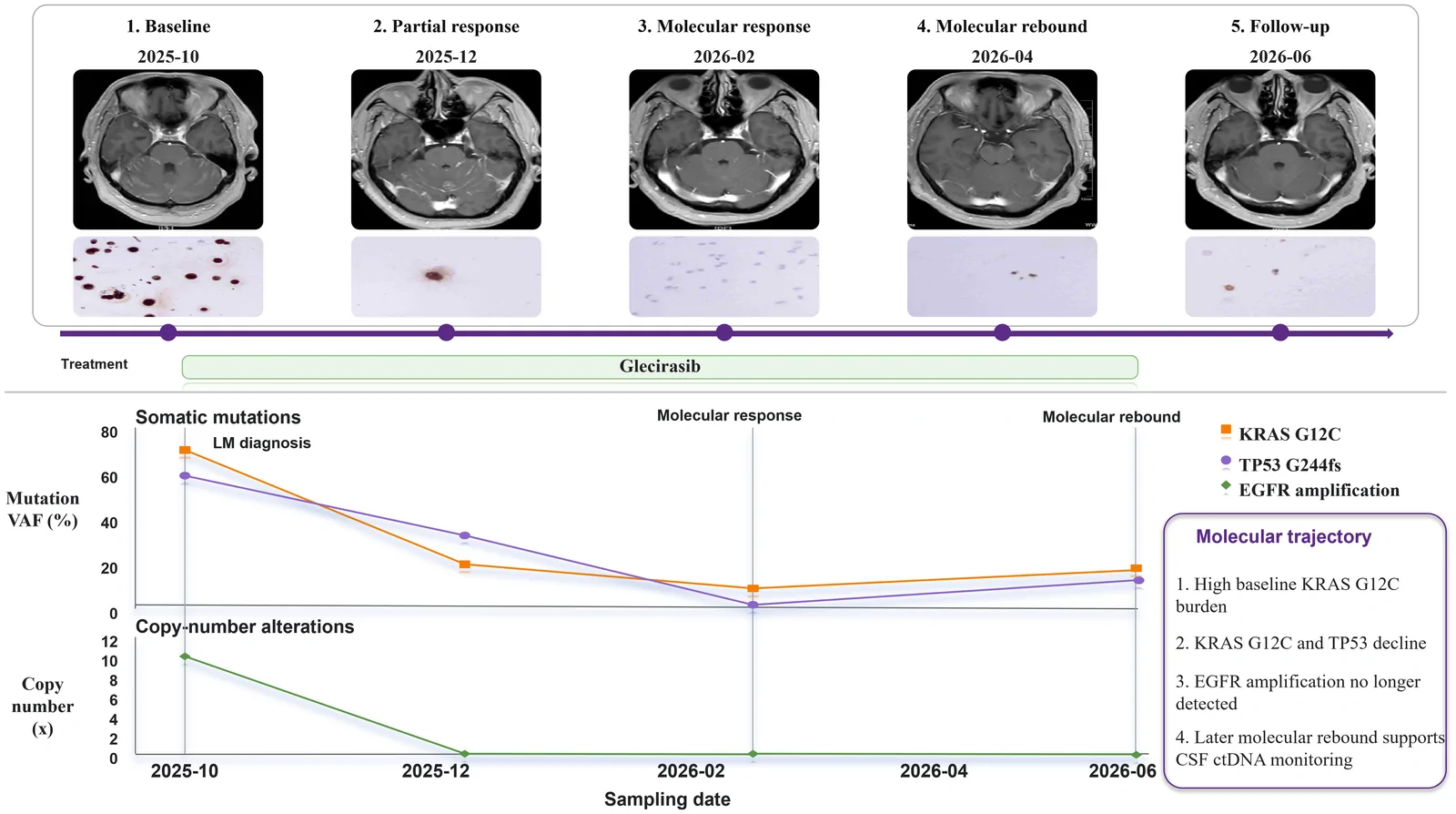
Primary KRAS G12C case (Case 6): clinical, cytological, imaging, and CSF ctDNA trajectory. All molecular values were obtained from CSF-supernatant cell-free DNA reports. Declining KRAS G12C and TP53 VAFs accompanied clinical/imaging improvement and cytological clearance; The exact historical assay tier at each time point was not recoverable; quantitative cross-time comparisons are descriptive.

## 4 Discussion

In this retrospective series of seven patients, glecirasib monotherapy was followed by LM disease control in six patients according to the study-defined integrated assessment. The best overall LM assessment was response in five patients, stable disease in one, and progressive disease in one; two patients subsequently developed LM progression during follow-up. No unequivocal extracranial progression was documented, and the best systemic response was stable disease in all seven patients. The difference between the LM and extracranial assessments is compatible with activity in the leptomeningeal compartment, although it does not establish preferential CNS efficacy.

The relevance of systemic *KRAS G12C* inhibition to LM cannot be assumed from activity outside the leptomeningeal compartment. LM develops within the CSF space, where drug exposure may be affected by the blood–CSF barrier and CSF flow, and tumor evolution may differ from that in extracranial disease or parenchymal brain metastases (8, 9). Consequently, neither an extracranial response nor activity in an intracranial tumor model establishes clinical activity in LM. Sotorasib and adagrasib have demonstrated systemic efficacy in previously treated *KRAS G12C*-mutant NSCLC (10–12), but prospective evidence in patients with active LM remains limited. Glecirasib has also shown systemic antitumor activity (6), and tumor suppression was observed in a preclinical intracranial lung cancer model (7). No CSF pharmacokinetic sample was available in the present series; therefore, the study cannot establish glecirasib CSF exposure or preferential CNS efficacy. The observations provide preliminary clinical evidence that complements, rather than confirms, preclinical intracranial findings.

The clinical background of this cohort is crucial for interpreting the observed control of LM. Prior to starting glecirasib treatment, all patients had developed LM following extensive treatment, characterized by a median ECOG PS of 3 and a median of 2 previous systemic treatment lines. Three patients had received fulzerasib and four had not received a prior KRAS G12C inhibitor. Outcomes were therefore reported separately: all three previously exposed patients had an LM response, whereas the four naive patients included two responses, one stable disease, and one progressive disease. These observations cannot establish retained sensitivity after prior KRAS G12C inhibition because subgroup assignment was nonrandomized, follow-up was immature, and on-target and bypass resistance mechanisms were not systematically profiled. During glecirasib therapy, no patients received concurrent anti-tumor treatments, minimizing confounding factors and strengthening the temporal link between glecirasib exposure and LM disease control. However, this does not establish causality. The small sample size, retrospective selection, absence of a control group, and variations in prior treatment history preclude reliable estimation of independent treatment effects. Despite these limitations, the findings suggest that glecirasib monotherapy may exhibit LM activity and advocate for standardized efficacy assessments in future prospective studies.

Serial CSF ctDNA monitoring adds a translational dimension to this case series. Because LM is anatomically and biologically linked to the CSF compartment, plasma ctDNA may incompletely capture CNS-specific disease biology (5). Prior studies have highlighted that CSF ctDNA can present a more representative genomic landscape of LM than plasma, with higher mutation detection rates, higher VAFs, and greater ability to detect copy-number alterations in CNS-compartment disease (13–17). In our previous large-scale study using serial CSF sampling through an Ommaya reservoir, a 20% change in CSF ctDNA maxVAF showed high concordance with clinical response assessment and provided prognostic value in lung adenocarcinoma with LM (17). That threshold was not applied in the present two-case descriptive series. In the present study, exploratory CSF ctDNA analyses complemented conventional LM assessment by identifying actionable molecular events and describing reported molecular changes during treatment. However, serial VAF changes were not interpreted as precise measures of tumor burden or as validated predictors of progression.

Serial CSF ctDNA was available in two patients and provided complementary information alongside conventional LM assessment. In the patient with acquired KRAS G12C, the alteration was identified in CSF after LM progression during EGFR-TKI treatment, supporting the rationale for glecirasib treatment. The subsequent decrease in KRAS G12C VAF was temporally concordant with symptomatic and imaging improvement. At later LM progression, KRAS G12C VAF increased, KRAS G12D emerged, and CDK4/MDM2 amplification increased. In this single patient, the pattern is compatible with, but does not establish, an acquired resistance mechanism. Previous studies have indicated that secondary KRAS variations and RAS-MAPK signaling reactivation may occur during the resistance development to KRAS G12C inhibitors (18–21). Additionally, copy number variations and bypass signaling may also play a role (21). In the patient with primary KRAS G12C, KRAS G12C and TP53 G244fs VAFs initially decreased and then increased at the final CSF assessment. No clinical, MRI, or cytological evidence of LM progression was present at that time. This molecular-clinical discordance should not be interpreted as evidence that a VAF rise predicts progression. Rather, it is an interesting exploratory observation that requires validation in larger, prospectively sampled studies. Existing studies advocate for the use of CSF ctDNA as an adjunct marker for assessing disease activity and prognosis (22, 23), yet they do not establish that isolated molecular elevation should warrant treatment discontinuation. Consequently, changes in CSF ctDNA must be interpreted alongside neurological examinations, CSF cytology, and CNS imaging (24).

The findings should be interpreted within the constraints of the study design. This was a single-center, retrospective case series of seven patients without a comparator, which limits both the precision and generalizability of the observed disease-control pattern. Prospective studies are needed to determine LM-specific efficacy and validate CSF ctDNA monitoring in this setting. The serial CSF ctDNA analyses were exploratory, and incomplete testing and sample-handling records at each time point precluded interpreting VAF changes as precise measures of tumor burden.

## 5 Conclusion

In this retrospective series of seven patients, glecirasib monotherapy was temporally associated with LM disease control in six patients, suggesting possible activity in KRAS G12C-positive NSCLC with LM. Exploratory serial CSF ctDNA analyses provided complementary molecular information in two selected patients.

## Data Availability

The original contributions presented in the study are included in the article/**Supplementary Material**. Further inquiries can be directed to the corresponding authors.

## Supplementary material

Supplementary Figure 1 Exploratory Kaplan-Meier curves for intracranial progression-free survival **(A)** and overall survival **(B)**, with censoring marks and numbers at risk.
