# Supplementary material for: Glecirasib treatment in KRAS G12C-positive non-small cell lung cancer with leptomeningeal metastasis
Source: Front Oncol. 2026 Sep 23;16:1956344. doi: 10.3389/fonc.2026.1956344 (PMC13642809; doi:10.3389/fonc.2026.1956344)
Supplement: Supplementary file 1 [file Table1.docx]

**Table S1.** Demographics, tumor characteristics, and prior treatment history

| **Case ID** | | **Smoking** **history** | **Histology** | **Stage at** **initial diagnosis** | | **Interval to LM** **(months)** | | **Neurological symptoms at LM** | **Prior** **immunotherapy** | **Prior** **chemotherapy** | **Prior anti-angiogenic** **therapy** | **prior intraventricular pemetrexed** |
| --- | --- | --- | --- | --- | --- | --- | --- | --- | --- | --- | --- | --- |
| Case 1 | Never | | Adenocarcinoma | IIB | 33 | | Dizziness, gait disturbance, visual disturbance, seizure | | No | Yes | Bevacizumab | Yes |
| Case 2 | Never | | Adenocarcinoma | IVB | 3 | | Headache, dizziness, nausea, vomiting, decreased visual acuity, and lower-extremity weakness | | No | Yes | Bevacizumab | No |
| Case 3 | Never | | Adenocarcinoma | IVB | 57 | | Headache, epileptic seizure, dysphagia, paralysis, and dysarthria | | Yes | Yes | No | No |
| Case 4 | Former | | Adenocarcinoma | IVB | 20 | | Headache, lower-extremity weakness | | Yes | Yes | No | No |
| Case 5 | Former | | Adenocarcinoma | IVB | 3 | | Headache, somnolence | | Yes | Yes | No | No |
| Case 6 | Former | | Adenocarcinoma | IVB | 12 | | Dizziness, headache, paresthesia of the limbs, tinnitus | | Yes | Yes | Bevacizumab | Yes |
| Case 7 | Former | | Adenocarcinoma | IVB | 22 | | Headache, diplopia, and fatigue | | Yes | Yes | Anlotinib | No |

Table S2. Leptomeningeal metastasis baseline characteristics

| **Case ID** | **LM diagnosis basis** | **CSF cytology** | **CSF protein (mg/dL)** | **CSF CEA (ng/mL)** | **Brain/spine MRI LM pattern** | **Serial CSF ctDNA monitoring** | **Glecirasib start date** | **Concomitant antitumor therapy during glecirasib** |
| --- | --- | --- | --- | --- | --- | --- | --- | --- |
| Case 1 | MRI + CSF cytology | Positive | 55.6 | 104.2 | Linear | Yes | 2025/12/7 | No |
| Case 2 | MRI | Negative | 84.3 | 0.257 | Nodular | No | 2025/9/12 | No |
| Case 3 | MRI + CSF cytology | Positive | 40.0 | 0.301 | Nodular | No | 2025/5/25 | No |
| Case 4 | MRI | Negative | 26.6 | <0.2 | Mixed | No | 2026/4/20 | No |
| Case 5 | MRI + CSF cytology | Positive | ND | ND | Mixed | No | 2026/4/2 | No |
| Case 6 | MRI + CSF cytology | Positive | 78.8 | 13.59 | Mixed | Yes | 2025/10/17 | No |
| Case 7 | MRI + CSF cytology | Positive | 23.4 | 1.31 | Linear | No | 2025/11/12 | No |

**Table S3. Summary of clinical responses**

| **Case** | **ECOG PS before** | **ECOG PS after** | **Neurological evaluation** | **Symptom evaluation** | **CSF cytology** | **CNS imaging** | **LM response** | **Best systemic response** |
| --- | --- | --- | --- | --- | --- | --- | --- | --- |
| 1 | 3 | 2 | Stable | Improved | Positive | Improved | Stable | SD |
| 2 | 3 | 2 | Improved | Improved | Negative | Improved | Response | SD |
| 3 | 4 | 4 | Worse | Worse | Positive | Stable | PD | SD |
| 4 | 2 | 2 | Improved | Improved | Negative | Improved | Response | SD |
| 5 | 3 | 1 | Improved | Improved | Negative | Improved | Response | SD |
| 6 | 3 | 1 | Improved | Improved | Negative | Improved | Response | SD |
| 7 | 3 | 2 | Improved | Improved | Negative | Improved | Response | SD |

Abbreviations: CNS, central nervous system; CSF, cerebrospinal fluid; ECOG PS, Eastern Cooperative Oncology Group performance status; LM, leptomeningeal metastasis; PD, progressive disease; SD, stable disease.

**Table S4.** Treatment-related adverse events by grade

| **Case ID** | **Grade 1** | **Grade 2** | **Grade 3** | **Grade 4** |
| --- | --- | --- | --- | --- |
| Case 1 | Anemia; hypertriglyceridemia | None | None | None |
| Case 2 | Nausea; diarrhea | None | None | None |
| Case 3 | None | Anemia; hypertriglyceridemia | None | None |
| Case 4 | Nausea | Hypertriglyceridemia | None | None |
| Case 5 | Anemia | Elevated blood bilirubin | Elevated AST; elevated ALT | None |
| Case 6 | None | None | None | None |
| Case 7 | Thrombocytopenia | Hypoalbuminemia | None | None |

**Abbreviations:** ALT, alanine aminotransferase; AST, aspartate aminotransferase; CEA, carcinoembryonic antigen; CSF, cerebrospinal fluid; ctDNA, circulating tumor DNA; LM, leptomeningeal metastasis; MRI, magnetic resonance imaging; ND, not determined

**Table S5. Patient-level outcomes according to prior KRAS G12C inhibitor exposure**

| **Prior G12C inhibitor** | **Case** | **Best LM assessment** | **iPFS, mo** | **iPFS status** | **OS, mo** | **Vital status** |
| --- | --- | --- | --- | --- | --- | --- |
| Yes (fulzerasib) | 2 | Response | 9.6 | Censored | 9.6 | Alive |
| Yes (fulzerasib) | 5 | Response | 3.1 | Censored | 3.1 | Alive |
| Yes (fulzerasib) | 6 | Response | 8.5 | Censored | 8.5 | Alive |
| No | 1 | Stable disease | 6.3 | Progression | 6.3 | Alive |
| No | 3 | Progressive disease | 2.9 | Progression | 3.5 | Dead |
| No | 4 | Response | 2.4 | Censored | 2.4 | Alive |
| No | 7 | Response | 7.7 | Censored | 7.7 | Alive |

**Clarifications regarding Tables S1-S4**

**Clarification 1:** All seven patients underwent baseline CSF cytology. Cases 2 and 4 had negative results; no cytology result was unevaluable.

**Clarification 2:** All CNS-directed treatments and systemic agents depicted in Figure 1 preceded glecirasib. No patient received concurrent intraventricular pemetrexed, radiotherapy, bevacizumab, or another antitumor agent during glecirasib. Exact cumulative intraventricular pemetrexed doses and dates were not consistently retrievable from the retrospective source record.

**Clarification 3:** For the grade 3 AST/ALT elevation in Case 5, dose-modification, resolution-time, and rechallenge details were not consistently retrievable; no grade 4-5 treatment-related adverse event was recorded.

**Clarification 4:** Longitudinal ctDNA values were transcribed from CSF-supernatant clinical laboratory reports. Exact assay tier by time point, pre-analytic variables, raw read-level filters, proprietary copy-number methods, and orthogonal validation were unavailable; quantitative cross-time comparisons are descriptive.
